# Aspergers – Different, Not Less: Occupational Strengths and Job Interests of Individuals with Asperger's Syndrome

**DOI:** 10.1371/journal.pone.0100358

**Published:** 2014-06-20

**Authors:** Timo Lorenz, Kathrin Heinitz

**Affiliations:** Department of Psychology, Freie Universität Berlin, Berlin, Germany; UNC Chapel Hill, United States of America

## Abstract

Rooted in the neurodiversity approach, this study provides an overview of the strengths and interests of individuals with Asperger's Syndrome. We interviewed136 individuals with Asperger's Syndrome and 155 neurotypical individuals via an online survey with regards to (a) demography, (b) occupational strengths, (c) general self-efficacy, (d) occupational self-efficacy, and (e) the job interest profile according to Holland. The vocational and educational fields of the individuals with Asperger's in the sample are more diverse than and surpass those classical fields stated in research and biographical literature. The comparison of both groups in cross-tables showed that the indicated strengths differ in several areas (Φ_Cramer_ = .02–.47), which means that a specific strength profile can be derived, and this profile goes beyond the clinical view of the diagnostic criteria. Individuals with Asperger's indicate lower self-efficacy, both general and occupational. Furthermore, a high concentration of individuals with Asperger's can be found in the areas I (Investigative) and C (Conventional) of Holland's RIASEC model.

## Introduction

The diagnostic concept of Asperger's Syndrome, which is part of the autistic spectrum, was introduced in the diagnostic manuals of both the APA and the WHO in the 1990s [1]. Since the introduction of this diagnosis and due to the improvement in diagnostics, many children have been diagnosed with Asperger's, which with the release of the DSM-V [2] has now become part of the autism spectrum disorder. Many of these children have by now completed school or university [3]. The school system is struggling to meet the needs of this group of people, and individuals with Asperger's still encounter obstacles upon entering careers. Individual biographies, interviews and media reports show that individuals with Asperger's work in professional fields such as research, IT, electrical engineering or mechanics [4], [5], [6]. To this day, however, no general survey exists to show which professional areas are frequented by employed individuals with Asperger's.

Several pragmatic approaches have been established to streamline the process of entering professional life for individuals with Asperger's, among them guidebooks [7] and organizations (e.g. “specialisterne” in Denmark, “Passwerk” in Belgium or “auticon” in Germany). These organizations function as intermediaries between individuals with Asperger's and businesses, yet the approaches still lack evidence-based tools to optimize the person-job-fit of individuals with Asperger's. Person-job-fit however affects job performance, turnover intentions, engagement and financial outcomes [8], [9]. It minimizes a person's boredom and anxiety while maximizing effort and enjoyment [8] and has a strong correlation with job satisfaction [10].

Progress in this field would be beneficial on two fronts: it would help individuals with Asperger's enter employment in jobs that fit their qualifications, all the while helping to meet the market's demand for qualified personnel.

The purpose of this study is to provide a primary overview of the professions, strengths and job interest profiles of individuals with Asperger's. This in turn will help lay the foundation for the development of approaches towards improving the occupational situation of individuals with Asperger's.

### Neurodiversity and strengths

Our approach is based on the theory of neurodiversity, a concept with footholds in neuroscience, evolutionary psychology and other fields, which considers autism a regular variant of the human brain [11]. This neuronal variance, which is regarded as natural, causes difficulties for individuals with Asperger's in areas such as empathy and social skills [12].

Regarding individuals with Asperger's as solely impaired or deficient would discount their strengths and capabilities [3], [13]. Their skills of concentration during long-lasting routine work, identification of logical rules and patterns, processing visual information, and the ability to remember facts, surpass neurotypical individuals [14]–[20] (a designation for persons with no divergent neurological development, in this case with no form of autism; [21]). These strengths can be an advantage in certain professions and thereby offer good prospects to integrate individuals with Asperger's into the professional world according to their abilities, creating a better person-job-fit. This is based on the strength philosophy, which assumes that persons can achieve more when they build on their strengths rather than try to balance their weaknesses [22].

Hence one aim of this study is to provide a summary of the task-relevant strengths (e.g. attention to detail, fine motor skills, logical reasoning, concentrativeness or visual skills) that individuals with Asperger's recognize in themselves, and to compare these with the descriptions of neurotypical individuals.

### Self-efficacy

Self-efficacy is another important intrapersonal factor that has an impact on several work-related outcomes. Among these are job performance [23], job satisfaction, intention to quit the profession [24] or career choice and development [25] and its predictive value for well-being and dealing with life's challenges [26]–[28]. Self-efficacy is defined as “belief in one's capabilities to mobilize the motivation, cognitive resources, and courses of action needed to meet given situational demands” [29]. Persons with high self-efficacy confront difficult situations with the certainty that they will remain in control [30]. Self-efficacy consists of two different facets, a generalized trait-like self-efficacy and a task-specific self-efficacy [31].

To our knowledge, no studies regarding individuals with Asperger's and self-efficacy have been conducted so far. Self-efficacy could be an explanation for a possible capacity-to-function-gap, i.e. that the available strengths (the capacity to function) of individuals with Asperger's are not made use of in (occupational) situations, leading to problems in the job or even to unemployment.

The social categorization of Asperger's Syndrome as a “disorder” or “disability” [13], and a high unemployment rate among this subgroup [32] has psychosocial effects such as low self-esteem, stress, social isolation [33], [34] and low self-efficacy.

This study will examine if individuals with Asperger's have lower general self-efficacy and a lower task-specific, in this case occupational, self-efficacy than neurotypical individuals. This yields two hypotheses:

1. *Individuals with Asperger's have a lower general self-efficacy than neurotypical individuals.*
2. *Individuals with Asperger's have a lower occupational self-efficacy than neurotypical individuals.*

### Job interest types

A work situation that matches the person's job interest type fulfills their psychological needs, leading to higher intrinsic motivation, attention, and job satisfaction [35]. Aside from their strengths, the professional interests of individuals with Asperger's will also be assessed in this study, since this will allow for an optimal identification and adaptation of the fit for specific professions.

The analysis of job interests will be based on Holland's RIASEC model [35] as a theoretical foundation. This model consists of six categories or interest types (Realistic, Investigative, Artistic, Social, Enterprising and Conventional). These classify persons according to their main interests (accordingly: manual, investigative, artistic, social, enterprising or organizing) and list specific prototypic professions for the respective types. Tests based on Holland's model generate a three-digit code out of the three interest types with the highest values.

The above-mentioned biographies, interviews and media coverage of individuals with Asperger's in professional fields such as research, IT and engineering lead to this study's hypothesis 3 for the model of job interest types:

1. *Individuals with Asperger's have a higher amount of interest type codes of one of the possible combinations in the categories R. I and C.*

## Method

### Participants and Procedure

This study has a total of 306 participants. Fifteen people were excluded from the data set (one due to implausible response behavior with no variance in answers, three on account of young age, two due to missing information regarding their Asperger's diagnosis, nine due to an AQ-10 score of <6 despite their declaration of an Asperger's diagnosis). The AQ-10 was employed to re-affirm the already existing diagnosis. It was by no means used as a diagnostic tool, which is why we did not exclude any neurotypical individuals. In total 291 persons were included in the analysis. Of these, 136 were individuals with Asperger's (86 women, 46 men, 4 other), between ages 18–65 (M_age_ = 35.54 years, *SD* = 10.59) and 155 were neurotypical individuals (91 women, 62 men, 2 other) between 18–60 years of age (M_age_ = 33.5 years, *SD* = 9.05). Participants were recruited by approaching group administrators on Facebook with the request to publish the link to the survey as well as by publishing the link in specialized internet forums. Individuals with Asperger's have time and again stated that they find online communication to be more comfortable than face-to-face communication [36]. Hence, this way of data acquisition was chosen in order to guarantee barrier-free access to the survey. The survey was administered in German. Participation was strictly voluntary, no compensation was supplied.

### Materials

#### Demographics

Participants were interviewed with regards to their country of residence, age, gender (“male”, “female”, and “other” – in order to accommodate individuals who do not identify with the gender binary), vocational training, college education, and current employment. The open input for current employment was encoded for analysis according to the Klassifikation der Berufe (Classification of occupations) 2010 [37], and the open input for college education was encoded according to the OECD [38].

**Strengths.** Participants were asked to pick one to five outstanding strengths from a list of 26 strengths (attention to detail, focus, team work, multitasking, numbers, repetitive tasks, creative solutions, systemizing, empathy, emotional control, physical work, fine motor skills stamina, consistency, flexibility, logical reasoning, concentrativeness, visual skills, auditory skills, apprehension, retentiveness, social skills, proactiveness, verbal skills). An open input-field allowed the participants to add their own concepts of strengths. Merely 136 of the 291 participants abided by the instruction to indicate one to five strengths. No difference between individuals with Asperger's and neurotypical individuals could be found in the compliance of this instruction, *Χ^2^* (1, *N* = 155) = 3.48, *p* = .06. Individuals with Asperger's (*M* = 6.65, *SD* = 3.70) named more strengths than neurotypical individuals (*M* = 5.69, *SD* = 2.74).

**Asperger's diagnosis.** Participants were asked whether they had received an official Asperger's Syndrome diagnosis. In addition, they completed the Autism Spectrum Quotient Test with 10 items (AQ-10) [39] with a 4-point scale, ranging from 1 = “*definitely agree*” to 4 = “*definitely disagree*” (e.g. “I find it difficult to work out people's intentions“). The test has an adult sensitivity of .88 and a specificity of .91, the cut-off was placed at 6 [39]. Cronbach's α of the AQ-10 was .89.

**General self-efficacy.** General self-efficacy was evaluated using the General Self-Efficacy scale (GSE) [40]. Participants responded to 10 items with a 4-point scale ranging from 1 = “*definitely agree*” to 4 = “*definitely disagree*” (e.g. “It is easy for me to stick to my aims and accomplish my goals.”). Cronbach's α was .90. We ran our analysis referring to the mean score of the GSE.

**Occupational self-efficacy.** Occupational self-efficacy was evaluated with the occupational self-efficacy scale [41]. Participants responded to 8 items with a 4-point scale ranging from 1 = “*definitely agree*” to 4 = “*definitely disagree*” (e.g. “I feel that I meet most occupational demands.”) Cronbach's α of this scale was .91. We ran our analysis referring to the mean score of the occupational self-efficacy scale.

**Job interest type.** Participants completed the revised German version of the General Interest Structure Test (AIST-R) [42]. Participants responded to 60 items with a 5-point scale ranging from 1 = “*I am not interested at all; I do not enjoy this at all*” to 5 = “*I am highly interested; I very much enjoy this*” (e.g. “Reading academic articles” or “Working with metal/wood, building something out of metal/wood”). The responses result in a three-digit code, which consists of a subsequent ranking of the six categories, from highest to lowest score. The three highest scores in this ranking then make up the code. Cronbach's α was .88. We ran our analysis referring to the standard scores of the AIST-R.

### Data analysis

The data was checked for the appropriate prerequisites to conduct our data analysis doing t-tests and Χ^2^-tests. Due to forced choice in the standardized questionnaires there was no missing data.

## Results

### Demographics

The largest part of the persons in the sample originated from Germany 93%), followed by Switzerland (2%) and Austria (1%). Altogether, 3% came from non-German speaking countries and 1% of the participants did not answer the question about their current country of residence.

Of the individuals with Asperger's, 55.9% stated to have absolved occupational training, as did 39.4% of neurotypical individuals. University degrees were held by 36.8% of individuals with Asperger's and 71% of neurotypical individuals. Table 1 relates the courses of study stated in the survey to the total amount of students in Berlin, Germany, in the winter semester of 2012/13 [43]. In comparison to the students in Berlin the number of individuals with Asperger's in this sample that state to be enrolled in social sciences (psychology, economics and business, educational sciences, sociology, law, political science, social and economic geography, media and communications) and natural sciences (mathematics, computer and information sciences, physical sciences, chemical sciences, earth and related environmental sciences and biological sciences) is disproportionately high. The number of neurotypical individuals in the social sciences is disproportionately high as well, whereas the number in natural sciences is disproportionately low. The corresponding numbers can be seen in table 1.

**Table 1:** The courses of study of the individuals in the sample vs. the total amount of students in Berlin, Germany in the winter semester of 2012/13.

	Humanities	Social Sciences	Agricultural Sciences	Medical and Health Sciences	Engineering and Technology	Natural Sciences	other
Students Berlin	24.61%	27.17%	2.49%	6.29%	21.84%	16.64%	0.98%
Aspergers	18.8%	39.6%	4.20%	6.30%	2.10%	29.2%	0.00%
NT	22.3%	57.6%	3.00%	2.00%	11.1%	4.00%	0.00%

Note: Aspergers  =  individuals with Asperger's in the sample, NT  =  neurotypical individuals in the sample.

44.9% of individuals with Asperger's stated to be currently employed. This number is lower than the information regarding employment given by the neurotypical individuals in this sample (71.6%), *Χ^2^* (1, *N* = 291) = 21.46, *p*<.001, Φ_Cramer_ = .27. Table 2 gives an overview of the open input about the current field of work. The majority of both groups can be found in the categories “health care, social affairs, and education” and “business organization, accounting, law and administration”. The comparison shows that more individuals with Asperger's work in the categories “production of raw materials“ and “natural sciences, geography and computer science”.

**Table 2:** Current employment of individuals with Asperger's vs. neurotypical individuals.

		Classification according to KldB 2010 Code									total
		Agriculture, forestry, animal husbandry, and horticulture	Production of raw materials	Construction, architecture, surveying, and building technology	Natural sciences, geography and computer science	Transportation, logistics, protection and security	Commercial services, retail, sales and distribution, hotels and tourism	Business organization, accounting, law and administration	Health care, social affairs, and education	Humanities, social sciences, and economic sciences, media, art, culture and design	
Aspergers	qty	0	6	0	6	1	2	12	21	4	52
	%	.0%	11.5%	.0%	11.5%	1.9%	3.8%	23.1%	40.4%	7.7%	100.0%
NT	qty	1	3	3	5	2	10	19	42	16	101
	%	1.0%	3.0%	3.0%	5.0%	2.0%	9.9%	18.8%	41.6%	15.8%	100.0%
total	qty	1	9	3	11	3	12	31	63	20	153
	%	.7%	5.9%	2.0%	7.2%	2.0%	7.8%	20.3%	41.2%	13.1%	100.0%

Note: KldB  =  Klassifikation der Berufe (classification of occupations), Aspergers  =  individuals with Asperger's, NT  =  neurotypical individuals, qty  =  quantity.

### AQ-10

In this sample the mean of the autism quotient scores for individuals with Asperger's is 8.86 (*SD* = 1.13), for neurotypical individuals it is 3.55 (*SD* = 2.90).

### Strengths

Cross-tables were generated to compare the distribution of the individual strengths in both groups. For an overview of the percent frequency of strengths for both groups and the results of the *Χ^2^* (1, *N* = 291) tests, see table 3. The level of significance was Bonferroni-Holm corrected. 16 of 26 strengths were reported differently comparing the two groups. The three strongest effect sizes could be found with empathy (Φ_Cramer_ = .47), attention to detail (Φ_Cramer_ = .39) and social skills (Φ_Cramer_ = .39). No relation of strengths with respect to the individuals with Asperger's AQ-10 score or gender could be determined.

**Table 3:** Frequency of indicated strengths of individuals with Asperger's vs. neurotypical individuals.

	%	%			
strength	Aspergers	NT	*Χ^2^*	*p*	Φ_Cramer_
Attention to detail	73	34	43.26	.000*	.39
Logical reasoning	60	35	18.86	.000*	.26
Reliability	49	44	0.63	.426	.05
Focus	48	17	30.91	.000*	.33
Systemizing	47	29	10.05	.002*	.19
Consistency	40	19	14.61	.000*	.22
Visual skills	36	18	12.02	.001*	.20
Creative solutions	35	26	2.65	.104	.10
Retentiveness	35	14	16.61	.000*	.24
Repetitive tasks	32	10	23.04	.000*	.28
Numbers	29	08	20.32	.000*	.26
Organizing ability	24	29	1.13	.288	.06
Apprehension	24	21	0.35	.553	.04
Verbal skills	24	41	9.45	.002*	.18
Auditory skills	23	05	21.32	.000*	.27
Stamina	22	20	0.19	.667	.03
Proactiveness	17	19	0.16	.690	.02
Fine motor skills	11	06	1.93	.164	.08
Concentrativeness	10	05	2.10	.148	.09
Emotional control	09	15	2.96	.085	.10
Physical work	09	08	0.02	.895	.00
Flexibility	04	26	24.91	.000*	.29
Social skills	04	35	43.52	.000*	.39
Multitasking	01	17	22.14	.000*	.28
Empathy	01	41	65.50	.000*	.47
Team work	00	25	39.52	.000*	.37

*  =  statistically significant after Bonferroni-Holm correction.

Note: Aspergers  =  individuals with Asperger's, NT  =  neurotypical individuals.

### Self-efficacy

Individuals with Asperger's reported a lower general self-efficacy (*M* = 21.44, *SD* = 5.32) than neurotypical individuals (*M* = 28.39, *SD* = 5.59), *t*(289) = −10.81, *p*<.001, *r* = .54. Furthermore, individuals with Asperger's also reported a lower occupational self-efficacy (*M* = 16.91, *SD* = 5.75) than neurotypical individuals (*M* = 22.72, *SD* = 5.20), *t*(289) = −9.05, *p*<.001, *r* = .47. These results are in favor of hypothesis 1 and 2.

The correlations between the statement of being currently employed and both self-efficacy scores were analyzed in an explorative data analysis. This showed the correlation with general self-efficacy to be statistically non-significant (*r* = .03, *p* = .70), and the correlation to occupational self-efficacy to be statistically significant (*r* = .26, *p*<.001) for individuals with Asperger's. Both self-efficacies, general self-efficacy (r = .20, *p* = .011) and occupational self-efficacy (*r* = .25, *p* = .002), show statistically significant relations with employment status for neurotypical individuals.

Furthermore, we tested whether or not a connection between the stated strengths of individuals with Asperger's and their self-efficacy scores exists. For the general self-efficacy 'score, visual skills (*r* = .27, *p* = 001) and proactiveness (*r* = .30, *p*<.001) proved to be statistically significant. For occupational self-efficacy, a statistically significant correlation with proactiveness (*r* = .30, *p*<.001) was found.

### Job interest type

Table 4 provides an overview of the group statistics results for the individual job interest types. Individuals with Asperger's score especially high in the interest types I (Investigative) and C (Conventional) and low in S (Social) and E (Enterprising). In order to test hypothesis 3 a cross-table was generated (see table 5). Individuals with Asperger's have a job interest code consisting of the types R (Realistic), I (Investigative) and C (Conventional) more often than neurotypical individuals, *Χ^2^* (1, *N* = 291) = 25.93, *p*<.001, Φ_Cramer_ = .30. Due to this data, results are in favor of hypothesis 3.

**Table 4:** Results t-tests job interest type scores – individuals with Asperger's vs. neurotypical individuals.

Interest	*M*	*SD*	*M*	*SD*	*M*	*df*	*t*	*p*
type	Aspergers	Aspergers	*NT*	NT	difference			
R	100.69	9.16	97.23	8.66	3.46	289	3.23	.001*
I	110.83	8.76	102.66	8.19	8.17	289	8.21	.000*
A	102.12	9.51	105.00	10.35	−2.88	289	−2.46	.014
S	90.56	12.94	102.11	12.83	−12.29	289	−8.12	.000*
E	87.76	10.22	102.86	11.45	−13.45	289	−10.51	.000*
C	110.32	10.32	102.88	9.80	7.44	289	6.30	.000*

*  =  statistically significant after Bonferroni correction.

Note: Aspergers  =  individuals with Asperger's, NT  =  neurotypical individuals, RIASEC refers to Holland's job interest types (R  =  Realistic, I  =  Investigative, A =  Artistic, S =  Social, E  =  Enterprising and C  =  Conventional) measured with the AIST-R.

**Table 5:** Comparison of RIC- and IC-Code – individuals with Asperger's vs. neurotypical individuals.

RIC-Code					total
			no	yes	
	Aspergers	quantity	91	45	136
		%	66.9%	33.1%	100.0%
	NT	quantity	141	14	155
		%	91.0%	9.0%	100.0%
total		quantity	232	59	291
		%	79.7%	20.3%	100.0%

IC-Code					total
			no	yes	
	Aspergers	quantity	32	104	136
		%	23.5%	76.5%	100.0%
	NT	quantity	113	42	155
		%	72.9%	27.1%	100.0%
total		quantity	145	146	291
		%	49.8%	50.2%	100.0%

Note: Aspergers  =  individuals with Asperger's, NT  =  neurotypical individuals, RIC and IC refers to Holland's job interest types (R  =  Realistic, I  =  Investigative and C  =  Conventional) measured with the AIST-R.

Due to the data and the high scores in the job interest types I and C of individuals with Asperger's, an explorative data analysis was conducted to determine the results of reducing the job interest code to these two interest types. A cross-table was generated and table 5 provides an overview of the percent distribution within the groups; the effect size in this analysis compared to the RIC-code analysis increases from a moderate to a relatively strong association [43], Φ_Cramer_ = .49, *Χ^2^* (1, *N* = 291) = 70.64, *p*<.001.

## Discussion

Results of this study show that the indicated occupational and educational fields of the individuals with Asperger's that participated in this study (Table 2) are more diverse than the hitherto existing literature [4], [5], [6] will have us believe, exceeding the fields of natural science, engineering, and IT. It becomes clear that future research and projects on the occupational integration of individuals with Asperger's generally can and should include more occupational areas than natural science, engineering and IT in order to better meet the needs of a diverse group of people, i.e. librarianship and fields of social science. When it comes to strengths, the data suggests that the strengths areas that were rarely indicated by individuals with Asperger's, i.e. empathy and flexibility, directly reflect the clusters of diagnostic criteria for Asperger's syndrome as provided by the DSM-IV [45] or, respectively, of autism spectrum disorder as provided by the DSM-V [2]. These criteria can result in a cluster of possible problems in everyday life [1]. The frequently indicated strengths, i.e. attention to detail or focus, form a cluster of their own, comprised of areas that, when combined, result in a very distinct strength profile. These strengths provide a perspective - beyond the clinical view - on areas in which individuals with Asperger's can draw on their strengths in order to fully tap into their potential within specific jobs.

Individual, tailored coaching could help to further a goal-oriented integration of individuals with Asperger's into the working world, drawing on available strengths while acknowledging problematic areas such as team work or social skills in face-to-face communication [46], [47]. Areas that require these exact strength profiles can be pinpointed within most occupational fields. Here, individuals with Asperger's could not just be integrated but might also be able to specifically show achievements superior to other candidates. Individual results on job-interests can further be used to determine corresponding occupational areas. Müller et al. [48] have shown that a high person-job-fit positively influences how individuals with Asperger's experience occupational life.

The data of this study shows that individuals with Asperger's have a lower general and occupational self-efficacy with a relation between employment and occupational self-efficacy. These results suggest that individuals with Asperger's could benefit from training programs that specifically target an increase in occupational self-efficacy. Generally, our findings concur with previous literature regarding individualized off-and on-the-job coaching for individuals with Asperger's that includes both the general aims of targeting job tasks, acclimation to the job site, and social integration [49], [50], [51], [52]. Additionally, a specific focus on occupational self-efficacy would bring further benefits.

### Future directions

Future research should attempt to replicate the data in the same context, as well as in other ethnic backgrounds, and with groups outside of the World Wide Web, in order to test and possibly increase the conclusions' generalizability.

Longitudinal studies and qualitative data can help to find the causes of the low self-efficacy scores, specifically of occupational self-efficacy. Furthermore, they could help to identify barriers in specific transition phases, i.e. the transition from school to vocational training or higher education, as well as entering and sustaining employment. The combination of these measures could help to develop programs for the transition to employment that would help to integrate individuals with Asperger's into the working world, and to accept them as full-fledged members of society.

### Limitations

Results of this study should be interpreted with the following limitations in mind. Firstly, participants were all recruited online. It is possible that findings may not generalize to people who are not using the internet or are not using social networks. The fact that more women participated in this study than would be expected when it comes to ASD could be explained by (a hypothetical) greater use of social media in women. More concerns about generalizability are warranted because this study used a nonprobability sample. Furthermore, participants were all of German-speaking descent, and were therefore relatively ethnically homogeneous. It is possible that individuals from other ethnic backgrounds would have reported different strengths or job interests. The participants were not diagnosed by means of a singular diagnostic method. Instead, they were asked to provide information about their Asperger's diagnosis. Due to the strong variation within the diagnostic process, we had to rely upon the participants' self-reported data of an existing diagnosis. Future research should include individuals with a confirmed diagnosis, possibly diagnosed by the same institution or at least using the same diagnostic process. This procedure would forgo self-reported data. Furthermore, it would provide insight into the differences within the spectrum by allowing for the comparison of individual scores.

The sample of neurotypical individuals shows a slight deviation from the results of the AIST-R reference sample. The aberration for the interest type „Realistic“ with a lower mean score could serve to explain the significant results in comparison with the surveyed individuals with Asperger's, because the mean score of individuals with Asperger's in the sample is equal to the mean score of the reference sample.

### Ethics statement

This study does not involve any conflict of ethics, since no clinical intervention was performed. Neither were blood or tissue samples taken for study purposes.

Participants were informed before participating that their responses would be treated confidentially and anonymously throughout and that all data would be analyzed in a generalized manner so that no conclusions could be drawn about individual persons.

Hence, we were not required to obtain approval from the ethics committee.

Furthermore, a consent form is not applicable, since an online survey was conducted. There was no contact between researchers and participants. The subjects participated voluntarily and were informed about the study's objectives and at all times giving their consent by filling out the online survey.
